# Performance adaptations to speed, agility, and quickness training in team-sport athletes: a systematic review and three-level meta-analysis

**DOI:** 10.3389/fphys.2026.1947365

**Published:** 2026-09-15

**Authors:** Ziyang Li, Hao Sun, Xuanji Luo, Minghui Li, Yiyuan Chong

**Affiliations:** 1 School of Sports Training, Wuhan Sports University, Wuhan, China; 2 Engineering University of the Joint Logistics Support Force, PLA, Chongqing, China

**Keywords:** athletic performance, speed, agility, and quickness training, systematic review, team sports, three-level meta-analysis

## Abstract

**Objective:**

This systematic review and three-level meta-analysis evaluated the effects of speed, agility, and quickness (SAQ) training on sprint, agility, and power performance in team-sport athletes and examined whether training characteristics and performance-test constructs moderated these adaptations.

**Methods:**

PubMed, Web of Science, EBSCO, ProQuest, the Cochrane Library, and Google Scholar were searched from inception to 1 July 2026. Randomised controlled trials involving team-sport athletes and SAQ interventions lasting at least 4 weeks were eligible. Three-level random-effects models accounted for dependent effect sizes, with cluster-robust variance estimation and the CR2 small-sample adjustment used for statistical inference. Subgroup analyses, meta-regression, and leave-one-study-out analyses were conducted. Risk of bias and certainty of evidence were assessed using the Cochrane Risk of Bias 2 tool and GRADE, respectively.

**Results:**

Sixteen studies were included. Sprint, agility, and power syntheses comprised 8 studies/17 effect sizes/344 athletes, 13/24/508, and 6/8/184, respectively. Pooled estimates favoured SAQ training for sprint (Hedges’ g = 0.53, 95% CI [0.15, 0.91]), agility (g = 0.77, 95% CI [0.42, 1.12]), and power (g = 0.60, 95% CI [0.06, 1.15]), with statistical significance retained after CR2 adjustment. However, all 95% PIs included the null. Favourable effects were observed for 5–10-m and 20–30-m sprint performance and pre-planned change-of-direction ability, whereas the effect on reactive agility was not statistically significant. No significant subgroup differences were found for sprint distance, agility construct, jump direction, sex, comparator type, or intervention duration. Neither intervention duration nor weekly training frequency showed a clear linear dose–response association. Agility findings were more robust than those for sprint and power. The certainty of evidence was low for agility and very low for sprint and power.

**Conclusions:**

Pooled average effects suggest that SAQ training may improve sprint, agility, and power performance in team-sport athletes, with the largest estimate observed for agility. Nevertheless, the inclusion of the null in all 95% PIs and the low-to-very-low certainty of evidence indicate uncertainty across training contexts and warrant cautious interpretation. SAQ training may therefore be considered one component of a comprehensive conditioning programme tailored to sport-specific demands. Further adequately powered randomised controlled trials with comprehensive training-dose reporting are needed.

**Systematic review registration:**

https://www.crd.york.ac.uk/PROSPERO/view/CRD420251273599, identifier CRD420251273599.

## 1 Introduction

Speed, agility, and quickness (SAQ) training is a multidimensional conditioning approach that systematically integrates short-distance sprinting, rapid acceleration and deceleration, multidirectional movement, change-of-direction running, high-frequency footwork, jumping, and stimulus-response drills. It is designed to enhance athletes’ rapid locomotor capacity, directional control, and neuromuscular function (Sheppard and Young, 2006; Paul et al., 2016; Uysal et al., 2025). Team sports such as football, basketball, and handball are characterised by intermittent bouts of high-intensity activity, requiring athletes to repeatedly perform accelerations, sprints, abrupt stops, changes of direction, jumps, and physical contacts with limited recovery (Bangsbo et al., 2006; Karcher and Buchheit, 2014; Stojanovic et al., 2018). Successful execution of these actions depends not only on linear sprint speed but also on braking and re-acceleration capacity, rapid force production, motor coordination, and perceptual–decision-making ability (Sheppard and Young, 2006; Paul et al., 2016). Because the sprinting, multidirectional movement, rapid footwork, and jumping components of SAQ training closely resemble the movement demands of team sports, this training modality has been widely incorporated into physical conditioning programmes and is considered to have the potential to enhance sprint, change-of-direction, and power performance.

In recent years, several studies have evaluated the effectiveness of SAQ training. Although some have reported improvements in sprinting, change-of-direction, jumping, and sport-specific movement performance (Jovanovic et al., 2011; Milanovic et al., 2013; Milanovic et al., 2014; Trecroci et al., 2016), the findings have not been entirely consistent. Training adaptations may be influenced by the type of sport, athletes’ age and training status, intervention duration, weekly training frequency, session duration, and the methods used to assess performance. In addition, some studies have labelled pre-planned change-of-direction tests as measures of “agility” without distinguishing between change-of-direction speed and reactive agility, the latter of which incorporates an external stimulus and a decision-making component. This conceptual conflation may lead to the aggregation of distinct performance constructs, thereby limiting the precision and practical relevance of the resulting conclusions (Sheppard and Young, 2006; Paul et al., 2016).

Previous systematic reviews and meta-analyses have generally supported the beneficial effects of SAQ training on sprint, change-of-direction, and jumping performance; however, several limitations remain within the current evidence base (Sun et al., 2025a; Sun et al., 2025b; Uysal et al., 2025; Ji et al., 2026). First, most studies have focused on football players, whereas evidence from other team sports remains comparatively limited, restricting the broader generalisability of the findings. Second, some reviews have primarily examined linear sprinting and pre-planned change-of-direction speed, with relatively limited synthesis of reactive agility and power outcomes. Consequently, potential differences in training adaptations across distinct performance constructs have not been fully investigated. Third, primary studies frequently report multiple sprint distances, change-of-direction tests, and jumping outcomes from the same sample. Because these effect sizes share participants and study-level characteristics, they cannot be assumed to be statistically independent.

To address these limitations, the present study conducted a systematic review and three-level meta-analysis to quantify the effects of SAQ training on sprint, agility, and power performance in team-sport athletes. In contrast to previous reviews, this study included athletes from a broader range of team sports, distinguished pre-planned change-of-direction speed from reactive agility, and incorporated multiple dependent effect sizes reported within the same study into a unified multilevel modelling framework. Potential moderating effects of sprint distance, agility construct, jump direction, comparator type, intervention duration, and weekly training frequency were also examined. The objectives were to determine the overall effectiveness of SAQ training, identify the performance capacities most likely to benefit, and clarify the conditions under which these adaptations may occur, thereby providing more targeted evidence to inform team-sport conditioning practice and the design of future intervention studies.

## 2 Methods

The review was designed and reported in accordance with the Preferred Reporting Items for Systematic Reviews and Meta-Analyses 2020 statement (PRISMA 2020) and was prospectively registered in the International Prospective Register of Systematic Reviews (PROSPERO; CRD420251273599) on 26 December 2025 (Page et al., 2021). The registration record is publicly available at https://www.crd.york.ac.uk/PROSPERO/view/CRD420251273599. The review was conducted in accordance with the prespecified protocol, with no deviations from the registered protocol.

### 2.1 Search strategy

PubMed, Web of Science, EBSCO, ProQuest, and the Cochrane Library were systematically searched from inception to 1 July 2026. The search strategy was developed according to the Population, Intervention, Comparator, Outcome, and Study Design (PICOS) framework and combined Medical Subject Headings (MeSH) with free-text terms. Search terms encompassed the concepts of “speed, agility, and quickness training” or “SAQ training”; “team sport” or “athlete”; and “sport performance”, “sprint”, “agility”, “change of direction”, “jump”, or “power”. Boolean operators (“AND” and “OR”) were used to combine the search terms. To maximise search sensitivity and comprehensiveness, supplementary searches were conducted in Google Scholar. The reference lists and forward citations of the included studies and relevant reviews were also screened to identify additional eligible records. The complete search strategies for all databases are provided in **Supplementary Material 1**.

All retrieved records were imported into EndNote 2025 (Clarivate, Philadelphia, PA, USA), and duplicate records were removed. Two reviewers independently screened titles and abstracts against the prespecified eligibility criteria and subsequently assessed the full texts of potentially eligible studies. Reasons for exclusion at the full-text stage were recorded. Any disagreements were resolved through discussion; when consensus could not be reached, a third reviewer adjudicated.

### 2.2 Eligibility criteria

Eligibility criteria were defined according to the PICOS framework.

Inclusion criteria were as follows: (1) Population (P): athletes participating in team sports, such as football, basketball, or volleyball; (2) Intervention (I): an SAQ training programme targeting speed, agility, and quick-response capabilities. The intervention was required to include at least two of the following components: linear sprinting or acceleration, pre-planned change-of-direction or agility drills, and stimulus-response or rapid-response exercises. The intervention duration had to be at least 4 weeks; (3) Comparator (C): usual training, small-sided games, another active training condition, or no intervention; (4) Outcomes (O): at least one measure of athletic performance, such as sprint performance, agility, reaction time, or power, with extractable data for the mean, standard deviation, and sample size; and (5) Study design (S): randomised controlled trials.

Exclusion criteria were as follows: (1) participants with sports injuries, medical conditions, or other health-related impairments; (2) qualitative studies, systematic reviews or meta-analyses, study protocols, non-peer-reviewed preprints, conference abstracts, or other non-full-text publications; (3) studies in which the prescribed intervention was not fully implemented or did not meet the predefined characteristics of SAQ training; (4) studies that did not report sufficient data for effect-size calculation; and (5) studies without a comparator group.

### 2.3 Data extraction and outcome coding

Two reviewers independently extracted data from the eligible studies using a standardised data-extraction form. Extracted information included study characteristics, participant characteristics, intervention features, comparator conditions, and outcome data. Any discrepancies arising during study selection, data extraction, or methodological appraisal were resolved through discussion after re-examination of the original report. When consensus could not be reached, a third reviewer adjudicated.

For studies with missing numerical data or results presented only graphically, the corresponding authors were contacted to request the required information. When no response was received, quantitative data were extracted from figures using WebPlotDigitizer (version 4.1; Ankit Rohatgi, Belmont, CA, USA). Studies for which sufficient data could not be obtained were excluded from the quantitative synthesis.

Before statistical pooling, two reviewers independently classified each outcome according to the underlying athletic-performance construct assessed by the test. Linear sprint tests were categorised as sprint performance. Tests involving a predetermined movement path were classified as pre-planned change-of-direction performance, whereas tests incorporating a visual or other external stimulus were classified as reactive agility. Horizontal jumping tests, such as the standing long jump, were categorised as horizontal power, whereas vertical jump tests, including the countermovement jump and other vertical jumping tasks, were categorised as vertical power.

When a study reported multiple eligible intervention groups, testing instruments, sprint distances, movement directions, or outcomes, all relevant effect sizes were retained. Their hierarchical dependency was recorded using a study-level identifier and a within-study effect-size identifier.

### 2.4 Risk-of-bias assessment

Risk of bias in the primary outcome results of the included randomised controlled trials was assessed using version 2 of the Cochrane Risk of Bias tool (RoB 2). The assessment covered five domains: bias arising from the randomisation process, bias due to deviations from intended interventions, bias due to missing outcome data, bias in outcome measurement, and bias in the selection of the reported result. Each domain, as well as the overall risk-of-bias judgement, was rated as “low risk”, “some concerns”, or “high risk”.

Two reviewers independently completed the assessments. Disagreements were resolved through discussion, with adjudication by a third reviewer when necessary.

### 2.5 Data processing and effect-size calculation

Sample sizes, means, and standard deviations were extracted for the intervention and comparator groups at baseline and post-intervention. When available, change scores and their corresponding standard deviations reported directly by the original studies were preferentially used. The mean change from baseline was calculated using **Equation 1**:

(1) $$\text{Δ}X=X_{post}-X_{pre}$$

where ΔX represents the mean change, and X_post_ and X_pre_ denote the post-intervention and baseline means, respectively. When the standard deviation of the change score was not reported, it was estimated using **Equation 2**:

(2) $${SD}_{\text{Δ}}=\sqrt{{SD}_{pre}^{2}+{SD}_{post}^{2}-2r\;{SD}_{pre}{SD}_{post}}$$

Where ***SDpre*** and ***SDpost*** represent the baseline and post-intervention standard deviations, respectively, and r denotes the within-participant correlation between pre- and post-intervention measurements. When r was not reported, a value of 0.50 was assumed for the primary analysis. To examine the robustness of this assumption, sensitivity analyses were additionally performed using alternative r values of 0.30 and 0.70. Standard errors and 95% confidence intervals were converted to standard deviations in accordance with Cochrane recommendations (Cumpston et al., 2019).

Because the included studies used different assessment instruments and measurement scales, continuous outcomes were expressed as standardised mean differences, with Hedges’ g used to correct for small-sample bias. The pooled standard deviation of the change scores and Hedges’ g were calculated using **Equations 3**–**5**:

(3) $${\text{SD}}_{\text{pooled,}\Delta }=\sqrt{\frac{({\text{n}}_{\text{SAQ}}-1){\text{SD}}_{\text{SAQ},\Delta }^{2}+({\text{n}}_{\text{CON}}-1){\text{SD}}_{\text{CON},\text{Δ}}^{2}}{{\text{n}}_{\text{SAQ}}+{\text{n}}_{\text{CON}}-2}}$$

(4) $$J=1-\frac{3}{4(n_{SAQ}+n_{CON})-9}$$

(5) $$g=J\times \frac{\text{Δ}X_{SAQ}-\text{Δ}X_{CON}}{{SD}_{pooled,\text{Δ}}}$$

where **J** is the small-sample correction factor. For outcomes in which higher values indicated better performance, the original effect direction was retained. For time-based outcomes in which lower values indicated better performance, the direction of the effect size was reversed. Accordingly, a positive Hedges’ g consistently indicated a more favourable change in athletic performance following SAQ training than under the comparator condition. Absolute effect sizes of approximately 0.20, 0.50, and 0.80 were interpreted descriptively as small, moderate, and large, respectively.

### 2.6 Statistical analysis

All statistical analyses were performed using R version 4.5.2 (R Foundation for Statistical Computing, Vienna, Austria) (Viechtbauer, 2010). All tests were two-sided, with statistical significance set at (p< 0.05).

#### 2.6.1 Primary-effect model

Because individual studies could contribute multiple effect sizes derived from different tests, sprint distances, movement directions, or intervention comparisons, the independence assumption underlying conventional two-level meta-analysis was unlikely to be satisfied (Assink and Wibbelink, 2016). A three-level random-effects model was therefore used to partition the total variance into sampling variance at level 1, variance amongst effect sizes within the same study at level 2, and variance between studies at level 3 (Cheung, 2019).

Models were fitted using the rma.mv function in the metafor package, with effect sizes specified as nested within studies. Variance components were estimated using restricted maximum likelihood.

To account for the limited number of studies and the statistical dependency amongst effect sizes within studies, cluster-robust variance estimation was additionally applied to the pooled-effect estimates, with study specified as the clustering unit. The Bell–McCaffrey CR2 small-sample adjustment and Satterthwaite approximation of the degrees of freedom were used to obtain robust standard errors, 95% confidence intervals, and (p) values (Tipton, 2015; Pustejovsky and Tipton, 2022).

#### 2.6.2 Heterogeneity, prediction intervals, and statistical power

Statistical heterogeneity was assessed using Cochran’s Q test, the within-study variance component (σ²_2_), the between-study variance component (σ²_3_), and the corresponding I² statistics. In the three-level model, within-study and between-study I² were calculated as the proportions of total variability (σ²_2_ + σ²_3_ + $\tilde{\text{v}}$) attributable to the respective variance components, where $\tilde{\text{v}}$ denotes the typical sampling variance. Total I² was calculated as the sum of the within-study and between-study components. I² values of< 25%, 25–50%, 50–75%, and > 75% were interpreted descriptively as indicating low, moderate, substantial, and considerable heterogeneity, respectively. In addition to the 95% confidence interval (95% CI), a 95% prediction interval (95% PI) was reported to indicate the range within which the true intervention effect would be expected to lie in a future comparable study. The statistical power of the pooled effect estimate for each outcome was evaluated using the ‘metameta’ package.

#### 2.6.3 Subgroup analyses and meta-regression

To further investigate potential sources of heterogeneity when I² exceeded 25%, subgroup analyses and meta-regression analyses were conducted. For sprint outcomes, subgroup analyses were performed according to sprint distance (5–10 m vs 20–30 m), comparator type (usual training vs other active comparators), and intervention duration (≤ 8 weeks vs > 8 weeks). For agility outcomes, subgroup analyses were conducted according to performance construct (pre-planned change-of-direction performance vs reactive agility), comparator type, intervention duration, and sex. For power outcomes, subgroup analyses were performed according to jump direction (horizontal vs vertical) and comparator type. Because comparator conditions varied across studies, comparator type was examined as a potential moderator of the intervention effect. The primary pooled models retained all eligible comparator conditions and were therefore interpreted as estimating average comparative effects across heterogeneous control conditions rather than effects relative to a single uniform comparator.

Between-subgroup differences were evaluated using the omnibus test of moderators from an intercept-containing moderator model, with the corresponding test statistic and p value reported. Statistical significance within an individual subgroup was not considered evidence of a significant difference between subgroups. Findings from subgroups comprising only a small number of independent studies were interpreted as exploratory.

For agility outcomes with sufficient data completeness and an adequate number of effect sizes, intervention duration in weeks and weekly training frequency were separately entered as continuous moderators in three-level meta-regression models. Meta-regression results were reported as regression coefficients (β), standard errors, 95% confidence intervals, and p values.

#### 2.6.4 Sensitivity analysis and assessment of small-study effects

Leave-one-study-out analyses were conducted to assess the robustness of the pooled estimates. When an individual study contributed multiple effect sizes, all effect sizes from that study were removed simultaneously before the model was refitted. Changes in the pooled effect estimate, 95% confidence interval, and heterogeneity estimates were then examined to determine whether the findings were materially influenced by any single study.

Contour-enhanced funnel plots were used to visually assess potential small-study effects. Egger’s regression test was performed when at least ten independent studies were available. Within the three-level modelling framework, the standard error of each effect size was entered as a moderator whilst retaining the clustered structure of effect sizes within studies. An Egger’s test result of p< 0.05 was interpreted as statistical evidence of funnel-plot asymmetry but was not considered, in isolation, definitive evidence of publication bias (Egger et al., 1997). When fewer than ten independent studies were available, funnel plots were assessed descriptively only.

### 2.7 Certainty of evidence

The certainty of evidence for the sprint, agility, and power outcomes was independently assessed using the Grading of Recommendations Assessment, Development and Evaluation (GRADE) framework. Evidence from randomised controlled trials was initially rated as high certainty and was downgraded, where appropriate, across five domains: risk of bias, inconsistency, indirectness, imprecision, and publication bias. The final certainty of evidence was classified as high, moderate, low, or very low.

Two reviewers independently completed the GRADE assessments. Disagreements were resolved through discussion or, when necessary, adjudication by a third reviewer.

## 3 Results

### 3.1 Study selection

The database searches identified 296 records, with an additional 30 records retrieved through reference-list screening and manual searching. After 121 duplicates were removed, 175 records underwent title and abstract screening, of which 127 were excluded because they were not relevant to the review question. The full texts of 48 articles were subsequently assessed for eligibility. Of these, 34 were excluded because they were systematic reviews or meta-analyses (n = 4), non-randomised studies (n = 8), did not report relevant outcomes (n = 11), or were unavailable in full text (n = 11), leaving 14 eligible studies.

Amongst the 30 records identified through additional sources, 28 were excluded following full-text retrieval and eligibility assessment, and two met the inclusion criteria. Overall, 16 studies were included in the systematic review and meta-analysis. The study-selection process is presented in **Figure 1**.

**Figure 1:**
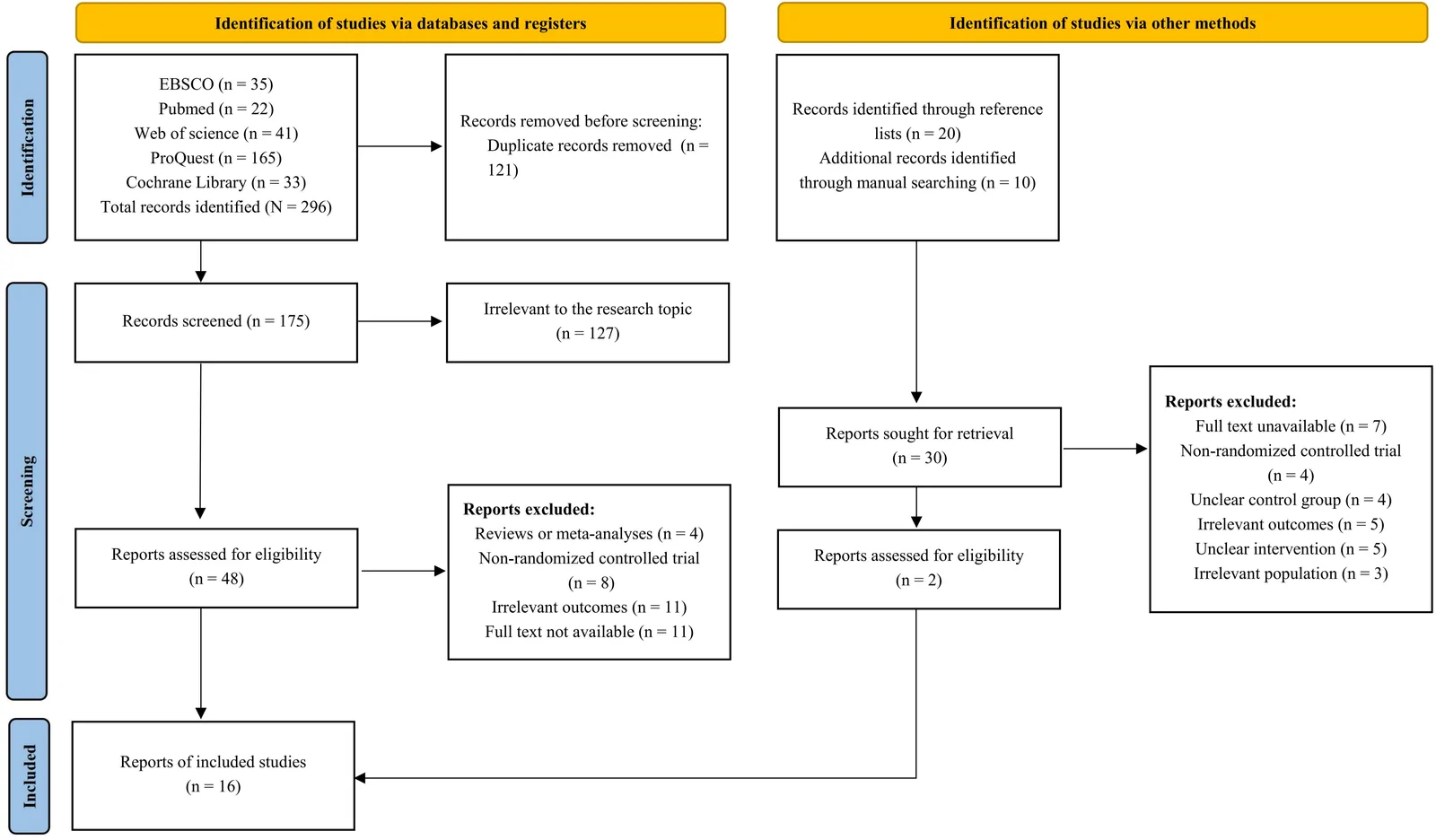
PRISMA flow diagram.

### 3.2 Characteristics of the included studies

Amongst the 16 included studies (Polman et al., 2004; Milanovic et al., 2013; Milanovic et al., 2014; Emeish1, 2015; Trecroci et al., 2016; Kusnanik et al., 2019; Moselhy, 2020; Anwer et al., 2021; Khaleel, 2022; Rengül et al., 2023; Singh et al., 2023; Suksai, 2023; Duan et al., 2024; Lee et al., 2024; Souilah and Kessouri, 2024; Lee et al., 2026), eight reported sprint outcomes, contributing 17 effect sizes from 344 athletes; 13 reported agility outcomes, contributing 24 effect sizes from 508 athletes; and six reported power outcomes, contributing eight effect sizes from 184 athletes. Because several studies reported more than one outcome, participant samples overlapped across outcome domains.

Sprint performance was primarily assessed using 5–10-m and 20–30-m linear sprint tests. Agility outcomes were classified as either pre-planned change-of-direction performance or reactive agility, whereas power outcomes comprised horizontal and vertical jump performance. Comparator conditions consisted mainly of usual training or other active training interventions.

The characteristics of the included studies are presented in Appendix 2. Statistical power plots for the pooled effects and funnel plots for the assessment of publication bias are shown in **Figures 2a–c** and **Figures 3a–c**, respectively.

**Figure 2:**
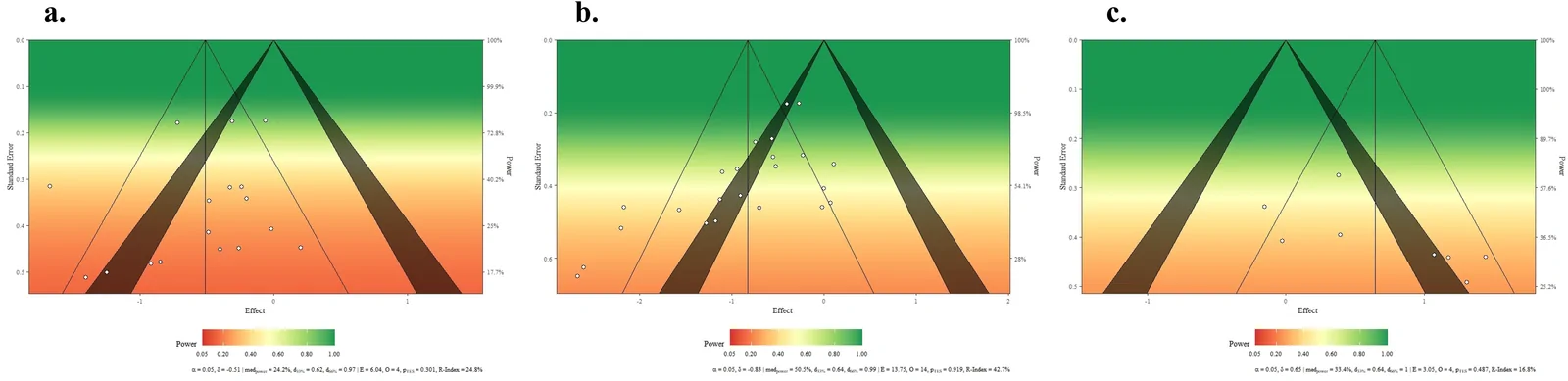
Statistical power plots for pooled effect sizes across outcomes. **(a)** Sprint; **(b)** Agility; **(c)** Power. Background shading indicates different levels of statistical power.

**Figure 3:**
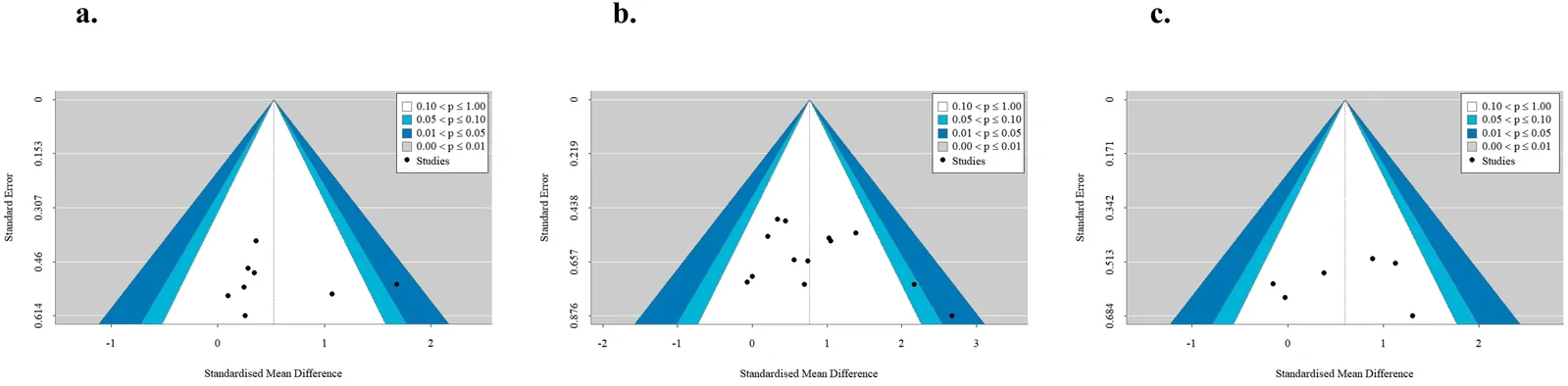
Contour-enhanced funnel plots for publication bias assessment across outcomes. **(a)** Sprint; **(b)** Agility; **(c)** Power.

### 3.3 Risk-of-bias assessment

The RoB 2 assessment indicated that one study was judged to have a low overall risk of bias, 13 studies raised some concerns, and two studies were rated as having a high risk of bias. The study by Emeish et al. was rated as high risk primarily because of concerns related to outcome measurement, whereas the study by Lee et al. (2026) was rated as high risk mainly because of missing outcome data. In the remaining studies, concerns were most commonly related to the randomisation process, deviations from the intended interventions, and selection of the reported results (**Figures 4**, **5**).

**Figure 4:**
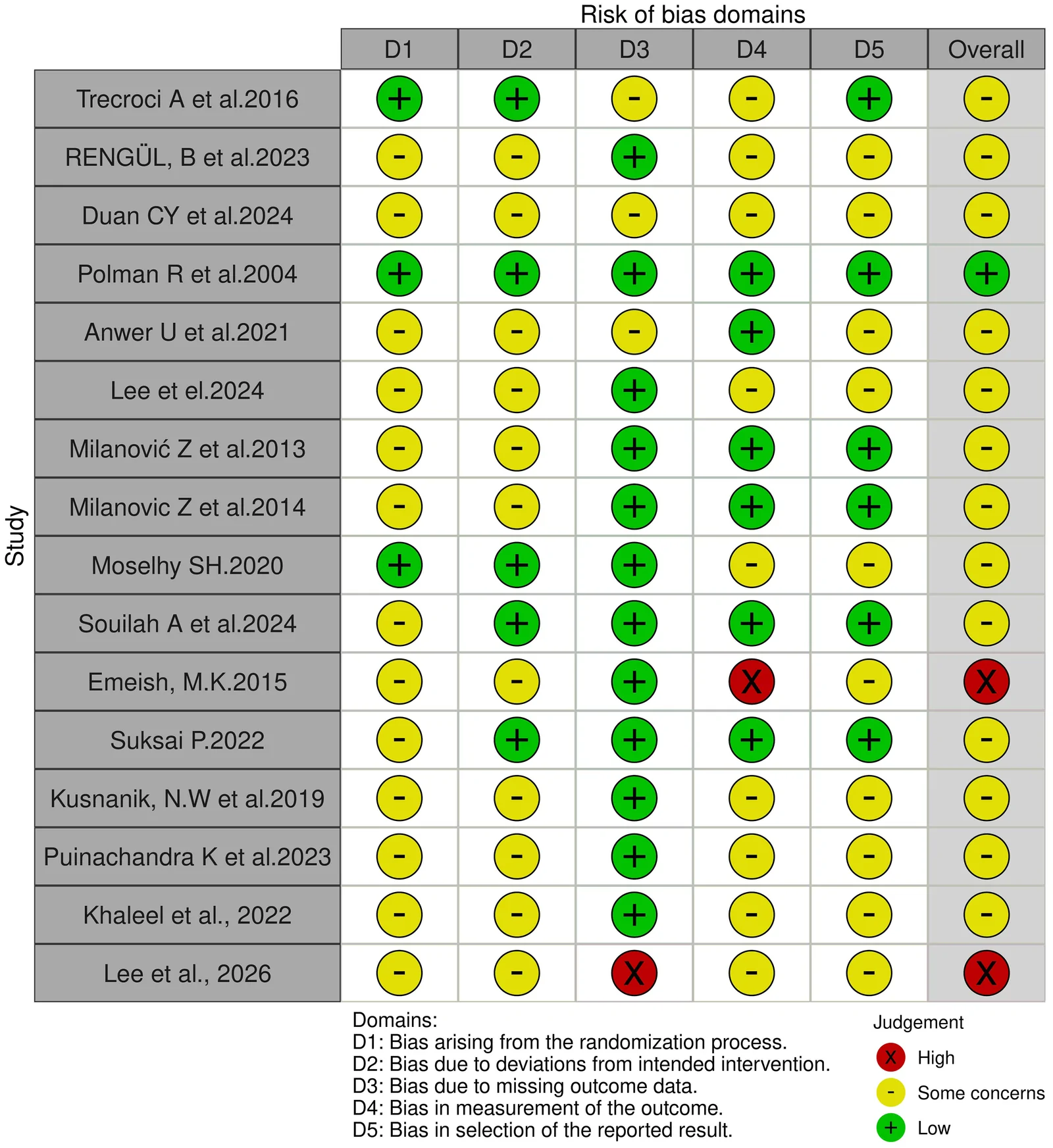
Risk of bias graph.

**Figure 5:**
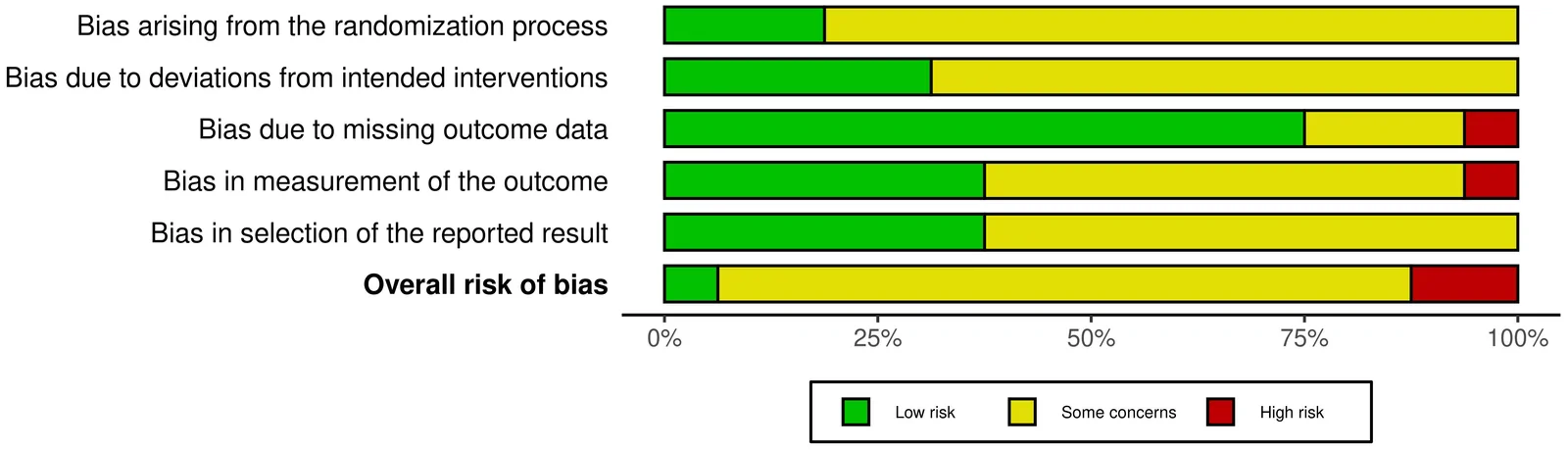
Risk of bias summary.

### 3.4 Meta-analysis

#### 3.4.1 Sprint performance

Eight studies contributed 17 effect sizes for sprint performance. The three-level random-effects model showed that SAQ training produced a statistically significant, moderate improvement in sprint performance (Hedges’ g = 0.53, 95% CI [0.15, 0.91], p = 0.010). The effect remained statistically significant after CR2 small-sample adjustment (95% CI [0.10, 0.96], p = 0.023). However, the 95% PI [-0.44, 1.49] crossed the line of no effect, indicating that the true effect may vary substantially across training contexts.

Significant heterogeneity was observed for sprint outcomes (Q = 43.925, p< 0.001), with a total I² of 53.81%. Within-study and between-study I² values were 16.93% and 36.87%, respectively, suggesting that heterogeneity was attributable primarily to differences between studies.

Subgroup analyses showed that SAQ training significantly improved both 5–10-m sprint performance (Hedges’ g = 0.56, 95% CI [0.12, 0.99], p = 0.017) and 20–30-m sprint performance (Hedges’ g = 0.50, 95% CI [0.08, 0.93], p = 0.022). However, the difference between sprint-distance subgroups was not statistically significant (p = 0.77).

When the comparator was usual training, SAQ training produced a significant improvement in sprint performance (Hedges’ g = 0.68, 95% CI [0.18, 1.18], p = 0.011). By contrast, the effect was not statistically significant when SAQ training was compared with other active training interventions (Hedges’ g = 0.29, 95% CI [-0.33, 0.92], p = 0.336). The between-subgroup difference according to comparator type was not statistically significant (p = 0.32).

For interventions lasting ≤ 8 weeks, SAQ training significantly improved sprint performance (Hedges’ g = 0.71, 95% CI [0.21, 1.21], p = 0.009), whereas the effect did not reach statistical significance for interventions lasting > 8 weeks (Hedges’ g = 0.29, 95% CI [-0.28, 0.86], p = 0.291). Nevertheless, the between-subgroup difference was not statistically significant (p = 0.26), providing insufficient evidence that shorter interventions were more effective than longer interventions (**Table 1**).

**Table 1:** Subgroup analyses for sprint.

Subgroup	K	Hedge’ s	95% CI	T-value	P_d_	I^2^-2	I^2^-3	P_b_
Sprint distance								0.77
5–10 m	8	0.56	[0.12, 0.99]	2.6945	**0.017**	15.93%	0.00%	
20–30 m	9	0.50	[0.08, 0.93]	2.5618	**0.022**	0.00%	65.36%	
Control type								0.32
RT	11	0.68	[0.18, 1.18]	2.8955	**0.011**	14.38%	53.00%	
Other active control	6	0.29	[-0.33, 0.92]	0.9933	0.336	0.00%	0.00%	
Intervention duration								0.26
≤ 8 weeks	10	0.71	[0.21, 1.21]	3.0075	**0.009**	0.00%	63.47%	
> 8 weeks	7	0.29	[-0.28, 0.86]	1.0935	0.291	38.11%	0.00%	

K, number of effect sizes; Hedges’ g, standardised mean difference; CI, confidence interval; Pd, p value for the pooled effect within each subgroup; Pb, p value for differences between subgroups; I²-2, within-study heterogeneity; I²-3, between-study heterogeneity; RT, routine training. Statistically significant results (p< 0.05) are shown in bold.

#### 3.4.2 Agility performance

Thirteen studies contributed 24 effect sizes for agility performance. The three-level random-effects model indicated that SAQ training significantly improved agility performance, with an effect of moderate-to-large magnitude (Hedges’ g = 0.77, 95% CI [0.42, 1.12], p = 0.0001). The effect estimate remained essentially unchanged after CR2 small-sample adjustment (95% CI [0.40, 1.14], p< 0.001), indicating that the overall finding was statistically robust. However, the 95% PI [-0.50, 2.04] suggested that the true effect may vary substantially across studies and training contexts.

Significant heterogeneity was observed for agility outcomes (Q = 79.936, p< 0.001), with a total I²of 66.49%. Within-study and between-study I² values were 52.24% and 14.26%, respectively, indicating that heterogeneity arose primarily from differences amongst tests or effect sizes reported within the same study.

When stratified by agility construct, SAQ training significantly improved pre-planned change-of-direction performance (Hedges’ g = 0.84, 95% CI [0.48, 1.19], p< 0.001), whereas the effect on reactive agility was not statistically significant (Hedges’ g = 0.41, 95% CI [-0.31, 1.12], p = 0.254). Although the estimated effects differed in magnitude, the between-subgroup difference was not statistically significant (p = 0.25).

When compared with usual training, SAQ training produced a significant large effect on agility performance (Hedges’ g = 0.99, 95% CI [0.53, 1.45], p< 0.001). In comparison with other active training interventions, the effect was smaller and did not reach statistical significance (Hedges’ g = 0.48, 95% CI [-0.05, 1.00], p = 0.073). The difference between comparator subgroups was not statistically significant (p = 0.14).For interventions lasting ≤ 8 weeks, SAQ training significantly improved agility performance (Hedges’ g = 0.92, 95% CI [0.46, 1.37], p< 0.001), whereas the effect did not reach statistical significance for interventions lasting > 8 weeks (Hedges’ g = 0.52, 95% CI [-0.09, 1.12], p = 0.090). However, the between-subgroup difference was not statistically significant (p = 0.286). Significant within-subgroup effects were also observed in both female athletes (Hedges’ g = 0.98, 95% CI [0.29, 1.67], p = 0.008) and male athletes (Hedges’ g = 0.70, 95% CI [0.31, 1.10], p = 0.001), with no significant difference between sexes (p = 0.48).

Meta-regression analyses showed no significant linear association between weekly training frequency and the effect of SAQ training on agility performance (β = 0.131, SE = 0.207, 95% CI [-0.274, 0.536], p = 0.526). Intervention duration was likewise not significantly associated with effect magnitude (β = -0.105, SE = 0.077, 95% CI [-0.257, 0.046], p = 0.173). Thus, the available evidence does not support a clear linear dose–response relationship for either weekly training frequency or intervention duration (**Table 2**; **Figure 6**).

**Table 2:** Subgroup analyses for agility.

Subgroup	K	Hedge’ s	95% CI	T-value	P_d_	I^2^-2	I^2^-3	P_b_
Agility type								0.25
Pre-planned COD	20	0.84	[0.48, 1.19]	4.8496	**< 0.0001**	23.80%	46.27%	
Reactive agility	4	0.41	[-0.31, 1.12]	1.1714	0.254	0.00%	0.00%	
Control type								0.14
RT	15	0.99	[0.53, 1.45]	4.4784	**0.0002**	68.47%	5.01%	
Other active control	9	0.48	[-0.05, 1.00]	1.8807	0.073	16.17%	1.84%	
Intervention duration								0.29
≤ 8 weeks	15	0.92	[0.46, 1.37]	4.1714	**0.0004**	68.64%	0.00%	
> 8 weeks	9	0.52	[-0.09, 1.12]	1.7740	0.090	23.90%	37.42%	
Sex								0.48
Female	9	0.98	[0.29, 1.67]	2.9381	**0.008**	66.87%	0.00%	
Male	15	0.70	[0.31, 1.10]	3.7021	**0.001**	0.00%	71.64%	

K, number of effect sizes; Hedges’ g, standardised mean difference; CI, confidence interval; Pd, p value for the pooled effect within each subgroup; Pb, p value for differences between subgroups; I²-2, within-study heterogeneity; I²-3, between-study heterogeneity; RT, routine training. Statistically significant results (p< 0.05) are shown in bold.

**Figure 6:**
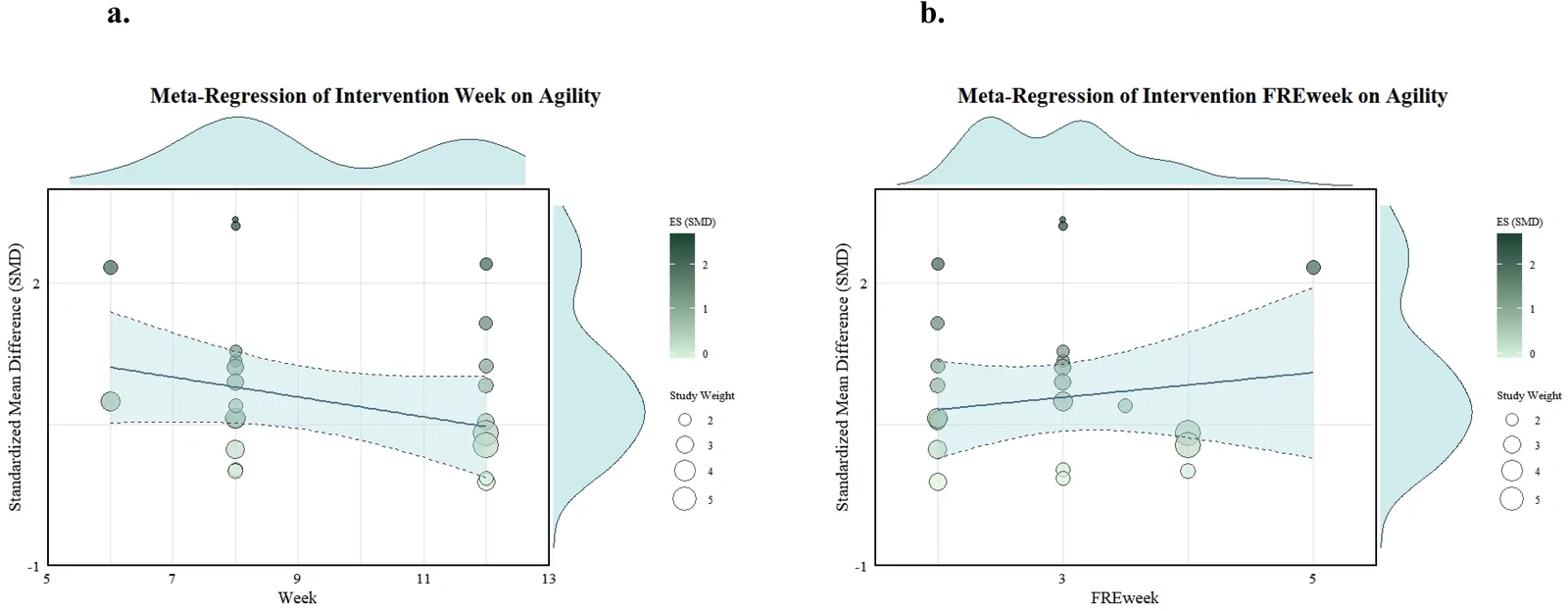
Meta-regression analyses of agility effect sizes by **(a)** intervention duration and **(b)** weekly training frequency. Bubble size represents study weight, colour intensity represents the standardised mean difference, solid lines indicate fitted regression estimates, and shaded bands indicate 95% confidence intervals.

#### 3.4.3 Power performance

Six studies contributed eight effect sizes for power performance. The three-level random-effects model showed that SAQ training significantly improved power performance, with an effect of moderate magnitude (Hedges’ g = 0.60, 95% CI [0.06, 1.15], p = 0.035). After CR2 small-sample adjustment, the effect remained statistically significant, although the result was close to the conventional significance threshold (95% CI [0.001, 1.20], p = 0.0497). The 95% PI [-0.65, 1.85] crossed the line of no effect, indicating that the true effect may vary considerably across training contexts.

Significant heterogeneity was observed for power outcomes (Q = 19.208, p = 0.008), with a total I² of 57.37%. Within-study I² was 56.71%, whereas between-study I² was only 0.66%, indicating that heterogeneity was attributable almost entirely to differences amongst power-related outcomes reported within the same study.

When SAQ training was compared with usual training, a significant improvement in power performance was observed (Hedges’ g = 0.86, 95% CI [0.28, 1.45], p = 0.011). By contrast, no significant effect was found when SAQ training was compared with small-sided games (SSG) training (Hedges’ g = −0.10, 95% CI [−0.98, 0.78], p = 0.798). The difference between comparator subgroups approached, but did not reach, statistical significance (p = 0.07).

When stratified by jump direction, the within-subgroup effects were not statistically significant for either horizontal jump performance (Hedges’ g = 0.70, 95% CI [-0.50, 1.89], p = 0.204) or vertical jump performance (Hedges’ g = 0.59, 95% CI [-0.09, 1.28], p = 0.078). No significant difference was observed between horizontal and vertical jump outcomes (p = 0.86). Detailed subgroup results are presented in **Table 3**.

**Table 3:** Subgroup analyses for power.

Subgroup	K	Hedge’ s	95% CI	T-value	P_d_	I^2^-2	I^2^-3	P_b_
Control type								0.07
RT	6	0.86	[0.28, 1.45]	3.6121	**0.011**	47.05%	0.00%	
small-sided games	2	-0.10	[-0.98, 0.78]	-0.2678	0.798	0.00%	0.00%	
Jump direction								0.86
Horizontal jump	2	0.70	[-0.49, 1.89]	1.4267	0.204	0.00%	52.14%	
Vertical jump	6	0.59	[-0.09, 1.28]	2.1207	0.078	0.00%	66.87%	

K, number of effect sizes; Hedges’ g, standardised mean difference; CI, confidence interval; Pd, p value for the pooled effect within each subgroup; Pb, p value for differences between subgroups; I²-2, within-study heterogeneity; I²-3, between-study heterogeneity; RT, routine training. Statistically significant results (p< 0.05) are shown in bold.

### 3.5 Sensitivity analyses

Leave-one-study-out analyses showed that the pooled effect estimates for sprint performance ranged from 0.375 to 0.580, with the direction of effect remaining unchanged across all analyses. After exclusion of the study by Anwer et al., the pooled effect was reduced to 0.375 (95% CI [0.000, 0.749], p = 0.050), indicating some attenuation in the statistical robustness of the sprint findings, although the direction of the overall effect remained favourable. The relatively greater influence of this study may partly reflect its distinctive comparison of SAQ training with specialised equipment versus SAQ training without equipment, together with the large between-group effect reported for 20-m sprint performance (Cohen’s d = 1.12). Moreover, all participants were inexperienced in SAQ training, which may have provided greater scope for early training adaptation.

The agility findings demonstrated comparatively strong robustness. After sequential exclusion of individual studies, pooled effect estimates ranged from 0.693 to 0.832, and none of the corresponding 95% CIs crossed zero. This indicates that no single study exerted a material influence on the overall conclusion.

By contrast, the findings for power performance were more sensitive to the exclusion of individual studies. Pooled effect estimates ranged from 0.470 to 0.744. After removal of the study by Polman et al., the effect was no longer statistically significant (Hedges’ g = 0.470, 95% CI [-0.036, 0.979], p = 0.070). A similar result was observed after exclusion of the study by Kusnanik et al. (Hedges’ g = 0.529, 95% CI [-0.038, 1.085], p = 0.070). Accordingly, the findings for power performance should be interpreted with caution.

Sensitivity analyses using alternative pre-post correlation coefficients produced findings consistent with those of the primary analysis across all outcome domains. Although the magnitude of the pooled effects varied with the assumed correlation coefficient, the direction and statistical significance of the effects remained unchanged, indicating that the overall conclusions were not materially influenced by the assumed value of r.

Detailed forest plots and sensitivity analyses for each outcome domain are provided in Appendix 3.

### 3.6 Certainty of evidence

The GRADE assessment indicated that the certainty of evidence was very low for sprint and power performance and low for agility performance. Downgrading was primarily attributable to the overall risk of bias across the included studies, substantial heterogeneity both between studies and amongst outcomes reported within individual studies, and imprecision in the effect estimates for sprint and power performance.

No further downgrading was applied for publication bias or indirectness because the available evidence did not indicate clear publication bias, and the study populations, interventions, and outcome measures were generally consistent with the review question. Only one included study was judged to have a low risk of bias, whereas 13 raised some concerns and two were rated as having a high risk of bias, supporting downgrading for risk of bias across all three outcome domains (**Table 4**).

**Table 4:** GRADE certainty of evidence.

Outcome	No. of participants (studies)	Risk of bias	Inconsistency	Indirectness	Imprecision	Publication bias	Other	Effect size [SMD (95% CI)]	Certainty (GRADE)
Speed	344 (Milanovic et al., 2013)	Serious	Serious	Not Serious	Serious	Not Serious	None	0.53 [0.15, 0.91]	Very Low
Agility	508 (Sun et al., 2025b)	Serious	Serious	Not Serious	Not Serious	Not Serious	None	0.77 [0.42, 1.12]	Low
Power	184 (Stojanovic et al., 2018)	Serious	Serious	Not Serious	Serious	Not Serious	None	0.60 [0.06, 1.15]	Very Low

The certainty of evidence according to the Grading of Recommendations, Assessment, Development, and Evaluations (GRADE) system is categorised into four levels: high, moderate, low, and very low. High certainty means we are very confident in the estimated effect, whilst moderate certainty indicates moderate confidence. Low certainty reflects limited confidence in the estimated effect, and very low certainty indicates very little confidence. This classification aids in assessing the reliability of research findings and guides the interpretation and application of evidence.

## 4 Discussion

This study used three-level random-effects models to synthesise statistically dependent effect sizes from 16 randomised controlled trials. The pooled estimates favoured SAQ training for sprint, agility, and power performance, with effect sizes of 0.53, 0.77, and 0.60, respectively, and remained largely unchanged after CR2 small-sample adjustment. The largest pooled effect was observed for agility, whereas the effects for sprint and power were moderate. However, the 95% PIs for all three outcomes crossed the line of no effect, and moderate-to-substantial heterogeneity indicated that the magnitude and consistency of the effects varied across studies and training contexts. Comparator conditions represented an additional source of variation, as SAQ training was evaluated against either usual training or other active interventions. Although effect estimates were consistently larger when SAQ training was compared with usual training, the between-subgroup differences were not statistically significant. Accordingly, the pooled estimates should be interpreted as average comparative effects across heterogeneous study and comparator conditions rather than as effects attributable exclusively to SAQ training. The magnitude of these effects may therefore vary according to athlete characteristics, sport, training programme, and comparator condition, and the findings should be interpreted cautiously given the low certainty of evidence for agility and very low certainty for sprint and power.

### 4.1 Effects of SAQ training on sprint performance

The pooled analysis yielded a moderate favourable effect of SAQ training on sprint performance in team-sport athletes, and the effect remained statistically significant after CR2 small-sample adjustment. However, the 95% PI crossed the line of no effect, and heterogeneity was driven predominantly by between-study differences, indicating that the magnitude and consistency of the effect may vary across training contexts. Although the direction of the pooled effect remained favourable in the leave-one-study-out analyses, its statistical robustness was attenuated after the exclusion of individual studies. Taken together, these findings suggest a possible benefit of SAQ training for sprint performance, but this effect should be interpreted cautiously given the very low certainty of evidence and the variability observed across studies.

These findings are broadly consistent with the existing literature. Sun et al. (2025b) reported that SAQ training improved 5–20-m sprint performance in football players, whilst a subsequent meta-analysis including athletes from multiple sports also demonstrated beneficial effects across several sprint distances (Sun et al., 2025a). Milanovic et al. (2014) observed improvements in short-distance sprint performance amongst youth football players following SAQ training, whereas Lee et al. (2024) reported more pronounced improvements over 20 and 30 m. Compared with these previous findings, the pooled effect in the present review was relatively conservative. This may reflect the broader range of sports included and differences in the statistical approach. In particular, the three-level model accounted for the dependency amongst multiple effect sizes reported within the same study, thereby avoiding the treatment of different sprint distances and testing conditions as statistically independent observations and producing a more cautious overall estimate.

The effects of SAQ training on sprint performance may be mediated by coordinated adaptations in neuromuscular control, force orientation, and movement technique. Sprint performance depends not only on the magnitude of lower-limb force production, but also on the athlete’s ability to apply ground-reaction force effectively in the horizontal direction within brief ground-contact periods (Samozino et al., 2011; Morin et al., 2012). Drills involving short accelerations, rapid step frequency, repeated acceleration, deceleration, and re-acceleration may enhance motor-unit recruitment, intermuscular coordination, body positioning, and the efficiency of horizontal force transmission. Similarly, jumping, bounding, and rapid landing drills repeatedly stimulate the stretch–shortening cycle and may improve pre-activation before ground contact and the transition from eccentric braking to concentric force production, thereby enhancing rapid force-generating capacity (Gollhofer et al., 2012). Nevertheless, most SAQ programmes rely predominantly on body-mass exercises and high-velocity movement tasks and may therefore provide a relatively limited stimulus for maximal-strength development and structural muscular adaptation. Accordingly, the observed sprint improvements may primarily reflect neural and technical adaptations, which may partly explain the moderate effect identified in this review.

Overall, SAQ training represents an effective strategy for improving sprint performance in team-sport athletes. Its effectiveness is nevertheless likely to depend on training intensity, the distribution of sprint distances, total training volume, and the quality of recovery. Given the very low certainty of evidence for sprint performance, further rigorously designed randomised controlled trials with comprehensive reporting of training dosage are required to strengthen these conclusions.

### 4.2 Effects of SAQ training on agility performance

The pooled analysis yielded a moderate-to-large favourable effect of SAQ training on agility performance. The estimate remained stable after CR2 small-sample adjustment and leave-one-study-out analyses, making agility the most statistically robust of the three outcome domains. Nevertheless, the 95% PI crossed the line of no effect, and heterogeneity arose predominantly from differences amongst outcome measures within the same study, indicating that the magnitude and consistency of the effect may vary across performance constructs and training contexts. Accordingly, although the pooled findings suggest a possible benefit of SAQ training for agility performance, they should be interpreted cautiously given the low certainty of evidence.

The pooled effect identified in the present study was larger than those reported by Sun et al (Sun et al., 2025a; Sun et al., 2025b), but was broadly comparable to the estimate for pre-planned change-of-direction speed reported by Ji et al. (2026). These differences may partly reflect variation in the conceptual definition of agility and the statistical methods applied. Previous studies have frequently used the term agility to describe pre-planned change-of-direction tests. In contrast, the present study distinguished pre-planned change-of-direction performance from reactive agility and used a three-level random-effects model to account for the dependency amongst effect sizes derived from different movement directions, courses, and testing conditions within the same study. This approach may provide a more precise estimate of adaptations across distinct performance constructs.

Subgroup analyses indicated that SAQ training significantly improved pre-planned change-of-direction performance, whereas the effect on reactive agility did not reach statistical significance. Pre-planned change-of-direction performance depends largely on deceleration and eccentric braking when approaching the turning point, trunk and centre-of-mass control, placement of the plant foot, and rapid re-acceleration after the directional transition (Brughelli et al., 2008; Paul et al., 2016). SAQ drills such as shuttle runs, cone drills, agility-ladder exercises, and multidirectional sprints are generally performed repeatedly along predetermined movement paths. These exercises may enhance movement proficiency, lower-limb neuromuscular coordination, and the efficiency of the braking-to-re-acceleration transition, thereby facilitating transfer to pre-planned change-of-direction tests.

Reactive agility, by contrast, incorporates both movement execution and perceptual–cognitive processing. Athletes must perform visual scanning, identify relevant stimuli, select an appropriate response, and continuously adjust their movement (Young and Rogers, 2014; Paul et al., 2016). When training predominantly involves predetermined routes and provides limited exposure to opponent movements, ball trajectories, or unpredictable visual and auditory cues, adaptations may be confined primarily to movement execution rather than to the coupling of perceptual information and motor responses. Young and Rogers (2014) found that small-sided games may enhance reactive agility through their decision-making demands, whereas pre-planned change-of-direction training primarily improves change-of-direction speed. Similarly, Trecroci et al. (2022) showed that SAQ training and small-sided games do not produce identical adaptations in physical and cognitive performance. Ball involvement may also influence the specificity of SAQ adaptations. Ball-free drills may place greater emphasis on movement velocity, braking–reacceleration mechanics, and neuromuscular coordination by reducing concurrent technical demands, whereas ball-based drills integrate these physical qualities with ball control and sport-specific coordination, potentially increasing the technical specificity of training. Milanovic et al. (2013) reported improvements in almost all agility measures performed both with and without the ball following SAQ training, whilst Moselhy (2020) found no between-group differences in non-ball speed, agility, and quickness but significant improvements in dribble agility and dribbling-skill performance. These findings suggest that ball-based and non-ball SAQ may provide complementary rather than interchangeable training stimuli, although direct comparative evidence remains limited.

It should be noted that the reactive-agility subgroup included only four effect sizes and yielded a wide confidence interval; therefore, the non-significant finding should be interpreted as reflecting limited evidence rather than evidence of ineffectiveness. Reactive agility involves perceptual–decision-making processes in addition to physical change-of-direction ability, and light-based and sport-specific human- or opponent-based stimuli may assess different performance components (Scanlan et al., 2014; Paul et al., 2016). Indeed, light-based and live-opponent reactive-agility measures showed little commonality (Scanlan et al., 2014), whilst improvements following small-sided-game training appear to be driven primarily by improvements in decision time rather than movement-response time (Young and Rogers, 2014). Future adequately powered randomised controlled trials should therefore directly compare reactive SAQ using light-based cues, reactive SAQ using human- or opponent-based cues, and pre-planned SAQ conditions whilst matching training dosage and movement demands. Reactive agility should also be assessed under standardised and sport-specific stimulus conditions, with decision time and movement-response time reported separately to distinguish perceptual–decision-making adaptations from changes in movement execution.

### 4.3 Effects of SAQ training on power performance

The pooled analysis yielded a moderate favourable effect of SAQ training on power performance in team-sport athletes. This finding is broadly consistent with previous meta-analyses (Sun et al., 2025a; Sun et al., 2025b). Intervention studies by Polman et al. (2004) and Jovanovic et al. (2011) also reported improvements in vertical jump, horizontal jump, and repeated-jump performance following SAQ training, suggesting that its adaptations may transfer to the capacity for rapid force production.

These effects may be mediated primarily by adaptations in stretch–shortening-cycle function and neuromuscular performance. Jumping, rapid bounding, short-distance sprinting, and deceleration–re-acceleration drills require the musculotendinous system to generate concentric force immediately after a rapid eccentric action. Repeated exposure to these demands may enhance pre-activation before ground contact, motor-unit recruitment, and intermuscular coordination, whilst improving eccentric-to-concentric coupling and the utilisation of stored elastic energy. Collectively, these adaptations may increase force production within a limited time frame (Markovic and Mikulic, 2010). High-velocity movement drills may also improve the rate of force development and coordination amongst the lower-limb joints, thereby providing a neuromuscular basis for enhanced power performance (Cormie et al., 2011).

No significant difference was observed between horizontal and vertical jump outcomes, suggesting that SAQ training may facilitate rapid force production across different movement directions. Horizontal jumping depends predominantly on horizontal impulse generation and hip-extension capacity, whereas vertical jumping places greater emphasis on vertical impulse and coordinated extension of the hip, knee, and ankle. Accordingly, practitioners should align training content with the directional demands of the target sport by appropriately integrating horizontal sprinting, vertical jumping, and multidirectional plyometric exercises.

SAQ training produced a more pronounced improvement in power performance when compared with usual training, suggesting that its high-velocity force-production and stretch–shortening-cycle demands may provide an additional adaptive stimulus beyond routine sport-specific practice. Overall, SAQ training may be incorporated as an important component of power development in team sports and combined with resistance and sport-specific plyometric training to promote the concurrent development of maximal strength and rapid force-producing capacity.

### 4.4 Practical applications and study limitations

The present findings suggest that SAQ training may concurrently enhance acceleration, braking, change-of-direction performance, and rapid force production through partly shared neuromuscular adaptations. Its primary value, however, lies in its integration within a comprehensive conditioning programme for team-sport athletes rather than as a substitute for resistance training, plyometric training, or sport-specific opposed practice. In applied settings, practitioners may combine linear acceleration, deceleration and change-of-direction drills at different angles, horizontal and vertical jumping exercises, and externally stimulated reactive tasks. The relative emphasis placed on each component should be adjusted according to the demands of the sport, playing position, and the athlete’s individual performance deficits. Accordingly, coaches may manipulate ball involvement according to the primary training objective, with ball-free SAQ placing greater emphasis on movement capacity and ball-based drills facilitating its integration with sport-specific technical execution. Although interventions lasting ≤ 8 weeks produced clearer effects for some outcomes, the available evidence does not demonstrate that shorter interventions are superior to longer programmes. Training duration should therefore be determined according to the competitive schedule, load–recovery balance, and principles of progressive overload.

Several limitations should be acknowledged. First, the number of included studies and the overall sample size were relatively limited. Some subgroups, particularly those examining reactive agility, horizontal power, and active comparators, contained few effect sizes, thereby reducing the statistical power to detect moderator effects. Second, the included studies differed in sport, age, competitive level, training content, training dosage, and outcome-assessment methods. Several primary studies also raised concerns regarding randomisation, intervention adherence, data completeness, and selective reporting. Although the three-level meta-analytic framework accounted for dependency amongst effect sizes within studies, it could not fully resolve the underlying clinical and methodological heterogeneity. Third, some studies did not report the standard deviation of change scores or the correlation between pre- and post-intervention measurements, requiring variance estimates to be derived using assumed parameters. Fourth, comparator conditions were not uniform across studies, with SAQ training being evaluated against usual training as well as other active interventions. These comparators may differ in training exposure and in their own capacity to elicit performance adaptations; therefore, the pooled effects should not be interpreted as reflecting the isolated effect of SAQ training alone. Although comparator-specific subgroup analyses were conducted, the limited number of studies within some comparator categories precluded firm conclusions regarding comparator-related effect modification. Moreover, the current evidence is based predominantly on short-term field-based assessments of sprinting, change-of-direction, and jumping performance and is therefore insufficient to establish the long-term retention of training adaptations or their transfer to competitive match performance.

## 5 Conclusion

The findings of this study suggest that SAQ training may benefit sprint, agility, and power performance in team-sport athletes, with the largest effect estimate observed for agility and moderate effects for sprint and power. Favourable effects were observed for both 5–10-m and 20–30-m sprint performance and for pre-planned change-of-direction performance, whereas the evidence for reactive agility remained inconclusive. No clear differences were identified according to sex, intervention duration, sprint distance, or jump direction, and neither intervention duration nor weekly training frequency showed a clear dose–response relationship.

Overall, SAQ training may be considered as one component of a comprehensive conditioning programme for team-sport athletes, with sprinting, change-of-direction, jumping, and reactive drills tailored to sport-specific demands and integrated with resistance and sport-specific training. However, the 95% prediction intervals for all three primary outcomes included the null, and the certainty of evidence was low for agility and very low for sprint and power. Accordingly, the magnitude and consistency of these effects remain uncertain across training contexts, and the findings should be interpreted cautiously. Moreover, because the available evidence is derived predominantly from short-term field-based assessments, it remains unclear whether these performance gains are retained over the longer term or translate to competitive match performance. Future high-quality randomised controlled trials should incorporate longer-term follow-up assessments to determine the durability of SAQ-induced adaptations and their transfer to competitive match performance.

## Data Availability

The original contributions presented in the study are included in the article/**Supplementary Material**. Further inquiries can be directed to the corresponding author.
