# Supplementary material for: Performance adaptations to speed, agility, and quickness training in team-sport athletes: a systematic review and three-level meta-analysis
Source: Front Physiol. 2026 Sep 15;17:1947365. doi: 10.3389/fphys.2026.1947365 (PMC13619520; doi:10.3389/fphys.2026.1947365)
Supplement: Supplementary file 1 [file Table1.docx]

| Library |  | N |
| --- | --- | --- |
| EBSCO | (Speed, Agility, Quickness training OR SAQ training OR SAQ exercises OR Speed, Agility, Quickness exercises) AND (performance OR physical performance OR sport performance OR athletic performance OR speed OR agility OR flexibility OR explosiveness OR reaction time OR Strength OR Endurance OR power) AND (Basketball OR soccer OR volleyball OR football OR handball OR team sports) | 33+2 |
| Pubmed | ((((Speed, Agility, Quickness training) OR (SAQ training)) OR (SAQ exercises)) OR (Speed, Agility, Quickness exercises)) AND (((((((((((((performance) OR (physical performance)) OR (sport performance)) OR (athletic performance)) OR (skill)) OR (speed)) OR (agility)) OR (flexibility)) OR (explosiveness)) OR (reaction time)) OR (Strength)) OR (Endurance)) OR (power)) AND ((((((Basketball) OR (soccer)) OR (volleyball)) OR (football)) OR (handball)) OR (team sports)) | 21+1 |
| Web of science | ((((TS=(SAQ training)) OR TS=(Speed, Agility, Quickness training)) OR TS=(SAQ exercises)) OR TS=(Speed, Agility, Quickness exercises)) AND (((((((((((((TS=(performance)) OR TS=(physical performance)) OR TS=(sport performance)) OR TS=(athletic performance)) OR TS=(skill)) OR TS=(speed)) OR TS=(agility)) OR TS=(flexibility)) OR TS=(explosiveness)) OR TS=(reaction time)) OR TS=(Strength)) OR TS=(Endurance)) OR TS=(power)) AND ((((((TS=(Basketball) OR TS=(soccer)) OR TS=(volleyball)) OR TS=(football)) OR TS=(handball)) OR TS=(team sports))) | 39+2 |
| ProQuest | ("Speed Agility Quickness training" OR "SAQ training" OR "SAQ exercises" OR "Speed Agility Quickness exercises") AND ("performance" OR "physical performance" OR "sport performance" OR "athletic performance" OR "speed" OR "agility" OR "flexibility" OR "explosiveness" OR "reaction time" OR "strength" OR "endurance" OR "power") AND ("basketball" OR "soccer" OR "volleyball" OR "football" OR "handball" OR "team sport*" OR "team-based sport*") | 163+3 |
| Cochrane Library | (Speed, Agility, Quickness training OR SAQ training OR SAQ exercises OR Speed, Agility, Quickness exercises) in Title Abstract Keyword AND (performance OR physical performance OR sport performance OR athletic performance OR speed OR agility OR flexibility OR explosiveness OR reaction time OR Strength OR Endurance OR power) in Title Abstract Keyword AND ("basketball" OR "soccer" OR "volleyball" OR "football" OR "handball" OR "team sport*" OR "team-based sport*") in Title Abstract Keyword - (Word variations have been searched) | 33+0 |
