# Supplementary material for: Performance adaptations to speed, agility, and quickness training in team-sport athletes: a systematic review and three-level meta-analysis
Source: Front Physiol. 2026 Sep 15;17:1947365. doi: 10.3389/fphys.2026.1947365 (PMC13619520; doi:10.3389/fphys.2026.1947365)
Supplement: Supplementary file 2 [file Table2.docx]

**Table S2.** Participant and SAQ intervention characteristics

| **Study** | **N** | **Sex** | **Age** | **Sport** | **Comparison** | **SAQ Training** | **W/F/T** | **Outcome** |
| --- | --- | --- | --- | --- | --- | --- | --- | --- |
| Trecroci A et al.2016 | 35 | M | 10.57 ± 0.62 | Soccer | EG = SAQ  CG = SSD | Hoop footwork drills; short COD sprint drills; Basic speed-ladder drills; multi-directional COD sprints; Advanced speed-ladder drills; reactive agility drills;  Reaction-based ladder drills; opponent-based agility drills | 12/2/25 | 5-m、20-m、MICODT、RAT |
| RENGÜL, B et al.2023 | 40 | M | CG:11.52 ± 0.5  EG:11.38 ± 0.49 | Soccer | EG = SAQ  CG = SSD | Hoop footwork drills; short COD sprints; Basic ladder drills; multi-directional COD sprints; Advanced ladder drills; reactive agility drills; Visual-reaction ladder drills; opponent-based agility drills | 8/2/25 | 5-m、20-m、MICODT |
| Duan CY et al.2024 | 56 | M | SAQ:16.3 ± 0.8 SIT:15.9 ± 0.9 | Soccer | EG = SAQ CG = SIT | Basic footwork drills; short COD sprint drills; Basic footwork and agility drills with ladder; Advanced footwork drills; multi-directional COD sprints with ladder; Combined basic and advanced agility and footwork training; Resistance training | 8/2/20 | IAT、ATT |
| Polman R et al.2004 | 36 | F | 21.2 ± 3.1 | Soccer | EG1 = SAQ EG2 = SAQE CG = RT | Sprint mechanics; Speed; Agility; Power; speed endurance | 12/2/60 | Agility, left foot、Agility, right foot、VJ、Horizontal power、Turning |
| Anwer U et al.2021 | 54 | M | EG:18.57 ± 0.5  CG:19.64 ± 0.9 | Soccer | EG = SAQE CG = RT | Running footwork drills; plyometric drills; agility pattern drills; coordination/ladder drills; short sprint drills; reactive agility drills; overspeed sprint training. | 6/3/60 | VJ、20-m sprint、IAT |
| Lee et el.2024 | 19 | F | 18.89 ± 0.80 | Football | EG = SAQ CG = RT | Running technique drills; agility ladder drills; resisted speed training; hurdle agility drills; slalom sprint drills;  sprint and shuttle run drills; reactive agility drills. | 8/3/40 | 5-m、10-m、20-m、  30-m、Arrowhead agility (L)、Arrowhead agility (R)、SEMO agility、Reactive agility |
| Milanović Z et al.2013 | 132 | M | U19 | Soccer | EG = SAQ CG = RT | Dynamic flexibility training; movement mechanics drills; coordination/footwork drills; agility running drills; reactive speed training; sport-specific agility drills. | 12/4/120-170 | Sprint with 90°turns、Sprint 180° turns、Sprint 4×5 m、Sprint with backward/forward running |
| Milanovic Z et al.2014 | 132 | M | U19 | Soccer | EG = SAQ CG = RT | Dynamic flexibility drills; speed mechanics drills; quickness/footwork drills; agility and soccer-specific drills. | 12/4/120-170 | 5-m、10-m、20-m |
| Moselhy SH.2020 | 25 | F | U10 | Basketball | EG = SAQ CG = RT ESG | Speed sprint drills; resisted/assisted sprint drills; agility and footwork drills; multi-directional COD drills; reactive agility drills; quickness drills; sport-specific dribbling drills | 12/3/90 | 20-m、IAT、SLJ、5-m |
| Souilah A et al.2024 | 24 | M | 15.45 ± 0.50 | Soccer | EG = SAQ  CG = SSG | Footwork drills; agility ladder drills; multidirectional COD sprints; slalom drills; reactive agility drills; partner-based reactive agility drills | 8/4/20 | 10-m、20-m、CODs、SJ、CMJ |
| Emeish, M.K.2015 | 20 | M | 13.12 ± 1.23 | Handball | EG = SAQ  CG = RT | SAQ training; multidirectional sprint drills; reactive agility drills; coordination/flexibility exercises; explosive strength drills; sport-specific movement drills | 8/3/70 | 30-m、RAT、flexibility、SLJ |
| Suksai P.2022 | 20 | M | EG=20.2 ± 0.6 CG=20.5 ± 0.5 | Soccer | EG = SAQ  CG = RT | Agility pattern drills; short multidirectional sprints; lateral sprint drills; COD drills | 8/14/NR | IAT |
| Kusnanik, N.W et al.2019 | 26 | M | NR | Soccer | EG = SAQ  CG = RT | Sprint training; COD agility drills; agility ladder drills; plyometric drills; reaction drills | 6/3/NR | VJ、SLJ |
| Puinachandra K et al.2023 | 30 | M | 18-25 | Football | EG = SAQ  CG = RT | resisted sprint drills; shuttle run drills; agility ladder drills; plyometric drills; hurdle hop drills; COD drills | 6/5/60 | 10×4 Shuttle Run |
| Khaleel et al., 2022 | 18 | M | 17-19 | Handball | EG = SAQ  CG = RT | Agility ladder drills; short sprints; multidirectional COD drills; reactive agility drills; sport-specific shooting drills | 8/3/NR | IAT |
| Lee et al., 2026 | 36 | M | 14.0-14.6 | Soccer | EG = SAQ  CG = NMT | ladder drills, skips, bounding, carioca, pro-agility, T-drill, reaction drills, mirroring, accelerators | 8/3/30 | CODs、CMJ、Agility |

**Note:** EG: Experimental Group; CG: Control Group; ESG: exploratory study Group; NR: No Report; SAQ: speed agility, quickness; SAQ(E): SAQ with Equipment; SSG: soccer-specific control; SSD: soccer-specific drills; SIT: Sprint Interval Training; RT: Regular training; FST: Free Sprint Training;NMT:Neuromuscular training; M: Male; F:Female; W: Week; F: Frequency; T:Time; 5-m: 5-meter sprint; 10-m: 10-meter sprint; 20-m: 20-meter sprint; 30-m: 30-meter sprint;	IAT: Illinois agility test; RAT: Reaction agility test; MICODT: Modified Illinois change of direction test; CMJ: countermovement jump; SJ: squat jump; SLJ: standing long jump;
