# Supplementary material for: Performance adaptations to speed, agility, and quickness training in team-sport athletes: a systematic review and three-level meta-analysis
Source: Front Physiol. 2026 Sep 15;17:1947365. doi: 10.3389/fphys.2026.1947365 (PMC13619520; doi:10.3389/fphys.2026.1947365)
Supplement: Supplementary file 3 [file Table3.docx]

**Table 5.Sensitivity analysis for assumed pre-post Pearson’s correlation coefficients.**

| Variables | r | SMD | 95%CI.lb | 95%CI.ub | T-Value | P |
| --- | --- | --- | --- | --- | --- | --- |
| **Speed** | 0.3 | 0.4533 | 0.1241 | 0.7825 | 2.9188 | 0.0100 |
|  | 0.5 | 0.5269 | 0.1464 | 0.9075 | 2.9355 | 0.0097 |
|  | 0.7 | 0.6546 | 0.1876 | 1.1216 | 2.9713 | 0.0090 |
| **Agility** | 0.3 | 0.6416 | 0.3470 | 0.9361 | 4.5055 | 0.0002 |
|  | 0.5 | 0.7692 | 0.4182 | 1.1202 | 4.5330 | 0.0001 |
|  | 0.7 | 0.9946 | 0.5448 | 1.4445 | 45737 | 0.0001 |
| **Power** | 0.3 | 0.4930 | 0.0218 | 0.9642 | 2.4738 | 0.0426 |
|  | 0.5 | 0.6025 | 0.0550 | 1.1501 | 2.6019 | 0.0353 |
|  | 0.7 | 0.7909 | 0.0882 | 1.4935 | 2.6616 | 0.0324 |

**Note.** Sensitivity Analysis for Assumed Pre-Post Pearson’s Correlation Coefficients. r, assumed pre-post Pearson’s correlation coefficient; SMD, standardized mean difference; 95% CI.lb, lower bound of the 95% confidence interval; 95% CI.ub, upper bound of the 95% confidence interval.

**Figure S1.** Forest plot of the pooled effect on sprint performance.

**Figure S2.** Leave-one-study-out sensitivity analysis for sprint performance.

**Figure S3.** Forest plot of the pooled effect on agility performance.

**Figure S4.** Leave-one-study-out sensitivity analysis for agility performance.

**Figure S5.** Forest plot of the pooled effect on power performance.

**Figure S6.** Leave-one-study-out sensitivity analysis for power performance.
